# “Take away the greed of the private landlord housing market … because **that** is killing people”: Examining the political economy of housing and health inequalities in four English coastal towns

**DOI:** 10.1016/j.socscimed.2025.118707

**Published:** 2025-10-23

**Authors:** Victoria J McGowan

**Affiliations:** Newcastle University, United Kingdom

**Keywords:** Health inequalities, Social determinants of health, Extractive industries, Housing, Neoliberalism, Coastal towns

## Abstract

Health inequities are avoidable and unjust differences in health arising from how society and the economy are structured. These inequalities are driven by vertical political and economic systems that prioritise economic growth over wellbeing. Yet, empirical research on health inequalities often focuses horizontally on people, places, and their inter-relationships. This research adopts a vertical lens to examine the structural processes driving geographical inequalities in health, framing housing as an extractive industry that contributes to ill-health and inequality. Drawing on comparative ethnography aross four English coastal towns, Hartlepool, Blackpool, Hastings, and Torbay, where deprivation, health and social inequalities are among the most severe in the country, the research involved 4-6 weeks of immersive fieldwork in each site during 2023. Data collection involved in-depth interviews, walking interviews, and focus groups with 140 people living/working in these towns, supported by participant observation, field notes, documentary analysis, and photography. This paper will focus on participants discussions around housing as a major determinant of poor health and inequalities in their towns. Findings highlight vertical mechanisms such as ‘Right-to-Buy legislation, extractive wealth practices of private landlords, substandard housing conditions, financial strain, and housing insecurity in driving physical and mental ill-health. By highlighting the intersection of housing, the political economy, and health inequalities this study demonstrates the importance of ‘scaling-up’ our analyses to identify vertical processes which undermine horizontal efforts to reduce inequalities in health. Further examination of these dynamics, covering other determinants of health, is necessary to support the development of policies that go beyond mitigating health inequalities but also tackle the vertical structures that sustain them.

## 1 Introduction

Health inequities are widely recognised as avoidable and unjust differences in health arising from how society and the economy are organised (Bambra, 2012; Lynch, 2019). Despite calls to investigate these vertical political and economic determinants of health, (Bambra et al., 2019; Raphael, 2015; Sayer and McCartney, 2021), empirical research often remains focused on horizontal analyses of people, places, and the relationships between them. Vertical approaches to researching health inequalities focus on the broader systemic and structural determinants of health such as the role of national and international political economies, government policies, and power relations that shape health outcomes across populations. Whereas horizontal approaches concentrate on the more immediate, localised, and individual-level factors which influence health such as people’s ability to access services, their neighbourhood environments, and health behaviours (Bambra et al., 2019). This vertical-horizontal framing is developed following Bambra et al. (2019) who advocate for ‘scaling-up’ analyses to account for political and economic drivers of health, situating inequalities within the broader structures of the political economy.

While previous literature on lay perspectives on health inequalities has described horizontal factors such as individual health behaviours, access to services, jobs, and housing (Davidson et al., 2006; Garthwaite and Bambra, 2017; Popay et al., 2003) there is emerging evidence demonstrating more complex and nuanced understandings of the inter-play between the vertical and the horizontal (Bernard et al., 2024; Mackenzie et al., 2017; Smith and Anderson, 2018). This shift is welcome as despite decades of research and policy recommendations targeting the horizontal conditions in which people live and work, health inequalities have widened, and life expectancy has declined (Rashid et al., 2021; Scambler, 2024). This suggests that horizontal approaches fail to understand or address the root causes, namely inequalities in income, wealth, and power (Marmot and Bambra, 2024; Sayer and McCartney, 2021). By maintaining a horizontal analytic lens, research overlooks how vertical processes create and sustain health inequalities (Bambra et al., 2019; Coburn, 2004; McCartney, 2022; Sayer and McCartney, 2021). This, in turn, obfuscates the development of policies capable of reducing inequalities and instead favours temporary mitigation strategies (Kelly-Irving et al., 2023; Sayer and McCartney, 2021). Indeed, frameworks such as the social and commercial determinants of health have drawn attention to how social conditions and corporate practices shape population health. However, these are often applied in reductive or siloed ways that obscure the structural forces underpinning these exposures (Frank et al., 2020; Maani et al., 2023).

In the UK, and other high-income countries, the economy facilities resource flows from poorer to wealthier places, limiting the effectiveness of policies aimed at improving conditions for disadvantaged places. Without addressing these economic flows such policies may yield only moderate or temporary benefits (Sayer and McCartney, 2021). This vertical, relational perspective which highlights how societal structures sustain health inequalities is largely absent from empirical research. Some scholars, however, are beginning to reveal the mechanisms by which resources are extracted from disadvantaged groups. Hatcher (2019), for example, exposes a profitable ‘poverty industry’ in the United States, where private firms and underfunded state governments exploit federal aid intended for vulnerable populations. This work demonstrates how poor families, abused children, the elderly, and disabled individuals are used as a source of revenue (Hatcher, 2019).

Schrecker et al. (2018) demonstrate how extractive processes impact health and exacerbate inequalities. They argue that profits from industries like fossil fuel extraction, sand mining, and land grabs for export crops could be redistributed to improve living conditions and health care systems. However, revenues from these industries rarely remain in the regions where they are generated, undermining local governments’ ability to create healthier environments (Schrecker et al., 2018). Moreover, they call for research to focus on where extraction occurs, for what purpose, the impact on health and inequalities, and the political economies that drive these processes (Schrecker et al., 2018). Indeed, Bambra et al. (2019) have argued that without ‘scaling up’ our analyses to focus vertically and advocate for systemic change, health inequalities research risks becoming complicit in maintaining a ‘health inequalities industry’. This critique underscores the importance of using research to challenge the structures that reproduce inequality, rather than merely documenting them (Bambra et al., 2019; Heath, 2007; Mackenbach, 2010). This paper, therefore, focusing on housing, builds upon a growing (Garnham, 2015; Smith and Stewart, 2024) but still relatively underrepresented body of empirical qualitative research that applies a vertical, political economy lens to health inequalities.

While there is a well-established literature on the impact of housing on health, this often focuses horizontally on the internal condition of the home, housing tenure and characteristics of places (Gibson et al., 2011). There is less research which centres housing in the political economy and highlights the mechanisms by which property serves as an extractive industry, facilitated by government legislation, which ultimately drives inequalities in health between poorer and affluent places (Aalbers and Christophers, 2014). Hochstenbach (2025) has called for a reorientation towards a political economy of housing and health, arguing that prevailing framings rooted in horizontal proximate or individual-level explanations obscure the deeper structural and institutional drivers of inequality (Hochstenbach, 2025). Housing is a key pathway through which the political economy shapes health. Beyond its material condition, housing is increasingly influenced by financial logics and policies that commodify property and enable wealth accumulation for some while displacing or impoverishing others (Aalbers, 2016; Fields, 2017; Madden and Marcuse, 2016). The financialisaton of housing has turned it into a site for profit extraction through mechanisms such as rent increases, insecure tenancies, benefit capture (where public housing support is redirected to landlords as private profit), and poor-quality conditions, all of which contribute to psychological stress, physical ill-health, and social dislocation (Fields and Uffer, 2016; Mason et al., 2024; Rolnik, 2013). These outcomes are not isolated local problems but the result of national policy decisions such as deregulation, welfare retrenchment, and austerity, which have contributed to widening health inequalities between places and populations (Marmot and Allen, 2020; McCartney et al., 2021). Indeed, within the commercial determinants of health literature, this vertically oriented, political economy approach is being applied to housing and health. Lacy-Nichols et al. (2023) argue that housing systems are deliberately structured to support capital accumulation at the expense of public health. Their analysis of housing in Australia echoes many of the dynamics described by participants in this study, including financialisation, policy-driven exclusion, and the extraction of wealth from economically marginalised groups (Lacy-Nichols et al., 2023). Therefore, this paper presents findings drawn from a larger dataset generated through a comparative ethnography of the north south health divide in England, a persistent regional pattern in which northern areas experience poorer health outcomes and shorter life expectancies than the south (Bambra, 2016). Within this broader dataset, participants discussed a wide range of explanations for spatial inequalities, including housing, which they located within vertical structures of inequality, making housing a critical lens through which to understand the persistence of health inequalities.

## 2 Methods

This article draws on qualitative data from a longitudinal mixed methods study examining regional health inequalities between the north and south of England. Ethnographic fieldwork was conducted by the lead author from April to December 2023 in four English coastal towns: two in the north Hartlepool and Blackpool, and two in the south Hastings, and Torbay. Comparative ethnography is particularly effective for uncovering mechanisms that sustain health inequalities by virtue of the methodological privilege of prolonged immersion within field sites but also, the explicit comparison of similarities and differences across places (Lewis and Russell, 2011; Orton et al., 2019; Simmons and Smith, 2019). This approach enables a vertical lens by identifying commonalities across geographically and culturally distinct places shaped by national and international political and economic forces. Ethnographic spatial comparison thus reveals how these forces interact with people and place to produce regional health inequalities (Shore and Wright, 2011).

### 2.1 Field site selection

The four towns were selected by comparing indices of deprivation (2019) among the 20 % most deprived local authorities in England, identifying northern and southern areas with similar levels of deprivation and other related factors (e.g. educational outcomes, economic activity, socio-economic classification) but differing life expectancies (Ministry of Housing Communities and Local Government, 2019). The four coastal towns, Hartlepool, Blackpool, Hastings, and Torbay were identified as sites which offered the opportunity to examine differences in health outcomes between similar places. While all four towns shared similar characteristics, (coastal location, high deprivation, geographically isolated) people living in the southern towns had an average life expectancy two years higher than their northern counterparts. This paper focuses on a key commonality across the four towns, the political economy of housing as a determinant of health, an issue which emerged inductively through the fieldwork. A detailed socio-historical account of these towns to enable comparison between them is beyond the constraints of this paper but, an overview of each town is provided in the online supplementary materials and summarised in Table 1.

### 2.2 Data collection

Ethnographic research was conducted in these towns between April and December 2023 with the author residing in each place for 4–6 weeks. A total of 140 people living and/or working in these towns participated in one-to-one/dyad interviews (n *=* 95), walking interviews (n = 3), or focus groups (n = 42) which were supplemented with participant observation, field notes, documentary research, and photography (see Table 2 for breakdown of data collection methods by field site). Multiple methods of data collection were used, not only to offer participants flexibility in how they engaged with the research, but also to support a more holistic understanding of place. The embedded ethnographic approach and drawing on a range of methods helps capture the interplay between material conditions, shared values, and everyday social relations that influence health through both local dynamics and broader political and economic contexts (Garnham, 2018). Participants were eligible to take part if they lived and/or worked in any of the four towns and were recruited via email (if publicly available), in-person interactions, community events, or via flyers, newsletters, and social media. A photographic diary on Instagram, also supported engagement and recruitment. Snowball sampling was also adopted as participants shared study information with colleagues, family and friends.

Interviews were conducted at locations chosen by participants, such as cafés, community centres, hotels, parks, homes, and workplaces. While demographic data were not formally collected as the focus was on place, participants often self-identified several demographic categories. At the start of each interview, participants typically mentioned their age, which ranged from 17 to 87 years old. Gender, ethnicity, social class, or sexual orientation was self-reported by only five participants. The sample consisted of residents (n = 73), who worked in the voluntary and charity sector (n = 34), in the public or private sector (n = 19), voluntary and charity sector non-residents (n = 4) and public or private sector non-residents (n = 10) (see Table 3 for breakdown by field site).

Participation was voluntary, confidential, and written consent was provided for all formal interviews and focus groups. The author identified herself as a researcher during informal conversations which were documented in field notes. A £20 voucher was provided to participants interviewed in their free time, while those interviewed during their working day who spoke in relation to their job role were not renumerated. Interviews and focus groups followed a biographical approach focusing on participants’ perceptions and experiences of where they lived or worked (Creswell and Poth, 2017). As an opening question participants were asked how long they had lived in the area and what it was like to live/work there. This approach led to a largely biographical and chronological account of their life with the author asking questions spanning vertical and horizontal explanations of inequalities such as area perceptions, migration, employment, housing, education, health, community cohesion and economic deprivation. Interviews were audio recorded and transcribed verbatim.

### 2.3 Data analysis

Transcripts and fieldnotes were anonymised before being thematically analysed using open coding to identify initial categories which were framed around the research question: *How do people living/working in coastal towns understand and experience health inequalities, and how to they explain regional differences in health outcomes?* Categories were developed separately for each town before merging related codes across the full dataset. Themes were then developed into clusters of related codes (Braun and Clarke, 2022). This approach enabled comparisons both within and across towns. Themes identified within the narratives covered housing, precarious employment, labour exploitation, privatisation, managed decline, territorial stigma, and (in)voluntary migration. For this paper, I focus on the theme of housing, findings on the other themes will be reported in subsequent papers. Relevant codes were analysed both within and across the four towns. This approach enabled the identification of shared narratives as well as place-specific differences. Housing narratives, however, showed relatively little regional variation. This paper, therefore, highlights housing-related commonalities, while drawing attention to contrasting narratives where they arise. To ensure anonymity, only the town name and a participant reference number will be provided as an identifier in quotes to allow comparison.

### 2.4 Researcher positionality

The author is a white British female contract researcher from a working-class background in the North East of England. Her lived experience of deindustrialisation and austerity includes witnessing friends’ and family’s economic migration and premature deaths, some of up to 40 years earlier than regional and national averages. These experiences shape her focus on social justice and economic redistribution. Acknowledging and using this lived experience deepens analysis, challenging dominant notions of legitimate knowledge and preventing the marginalisation of alternative ways of understanding the social world (Bourdieu, 1977).

## 3 Findings

Participants across all four towns discussed wealth extraction as a mechanism driving health and social inequalities, highlighting the flow of resources from poorer to more affluent areas. These vertical explanations emerged inductively during fieldwork, adding further depth to existing qualitative research into lay explanations of health inequalities. Building on Smith and Stewart’s (2024) synthesis of lay accounts in Scotland, which identified local profiteers such as landlords and private companies as key actors in extracting resources from disadvantaged communities, this paper explores how similar dynamics are experienced in four English coastal towns (Smith and Stewart, 2024). Participants in this study situated localised understandings of deprivation and health inequalities within broader political economic contexts, describing how local places are deprived of resources through housing markets, privatised services, labour exploitation, and regeneration or disinvestment. While these themes will be explored in detail in future publications, this paper focuses on housing, specifically the impacts of Right-to-Buy legislation, private landlords, and houses of multiple occupation (HMOs).

### 3.1 Right to buy

The ‘Right to Buy’ (RTB) policy, introduced by the Thatcher government in the 1980s, allowed social housing tenants to purchase their homes at discounted rates (Eardley, 2022). While initially benefitting tenants committed to their communities by offering secure tenure and property wealth (Murie, 2016), the policy’s impact shifted over time. Some tenants sought council tenancies solely for RTB discounts, later selling to move elsewhere (Murie, 2016). Although RTB enabled some lower-income households to achieve homeownership, an estimated 40 % of RTB homes are now privately rented (Pattison and Cole, 2020). Private landlords increased from 61 % in the 1990s to 89 % by 2011 (Ronald and Kadi, 2018). Councils struggled to replace sold homes due to the withdrawal of funding for social housing, with investment redirected to private housing associations (Naqvi et al., 2021). Participants linked RTB to housing challenges, citing reduced social housing and the rise of private landlords.

“We have a lot of private landlords that- a lot of these street houses now are owned by private landlords. I blame Maggie Thatcher for that. Because we had a good council system. And people looked after their house, they were happy in their houses. The council kept up with repairs, and everything. And then, they were told they could buy them. So, a lot of them did. But then, some of the houses had subsidence, according to the council. And the council bought quite a few back. But the people were still allowed to live in them. But when they passed away, they were sold on. And a lot of them are private landlords now. And they’re not looked after as well” (Hartlepool 1).

Participants highlighted how the RTB policy transferred council housing into the private rented sector, contributing to declining housing quality. Councils had maintained housing stock to a high standard, but many RTB purchasers, particularly low-income buyers, struggled to afford repairs, leading to deterioration (McCall et al., 2020). Private landlords who acquired former council homes often neglected maintenance to maximise profits. Consequently, RTB reduced affordable housing availability and quality, and entrenched wealth extraction in the housing sector.

“I hate to blame Thatcher, but 4.9 million homes went in the Right to Buy, 4.9 million homes, and 20 % of the housing market evaporated overnight. So, straightaway, and again, it’s the idea that free market capitalism can work for working-class people. It doesn’t, and that’s why they’re called ‘working-class’ because of the labour market that creates the infrastructure to build it, do you know what I mean. The thing, as well, like, the needs of the whole- You know, the Right to Buy was to get more money into the economy, wasn’t it? That’s basically what it was used for, so it could give it more economic pressure. The thing is, even people who went and did the Right to Buy, across this country now there will be people sat in houses that got them out on the Right to Buy, that have kids sat in them that can’t afford the deposit to buy the house” (Blackpool 1).

“From what I can gather, there was a lot of social housing that got bought privately quite a while ago, and that obviously added to the problem” (Torbay 1).

Many participants viewed RTB as a *“disaster”* requiring national political intervention to address housing challenges. While some advocated for large-scale social housing construction, others highlighted physical barriers in coastal towns such as limited land, challenging terrain, and risks from coastal erosion, (Beatty et al., 2008; Fiorentino et al., 2024), making such developments difficult.

“So there has been political failure to address the agenda of housing. I also think Right to Buy has been a disaster, especially in the south-east where quite a big population in the south-east, in the Greater London area, and a lot of the stock which was council housing has gone. It has gone into the private sector. I know that the Scottish Government have abolished Right to Buy north of the border. I think any government- If there is an incoming Labour government, cross fingers and cross toes and all the rest of it, but there has to be an abolition of Right to Buy, plus a massive building programme. That is the only way that we are going to even get anywhere near this” (Hastings 1).

### 3.2 Private landlords

Privately rented accommodation, much of it formerly part of the social housing stock sold under Right to Buy, was discussed extensively in all four towns. Participants viewed landlords negatively, seeing them as profiting from the propertyless and public subsidy via housing benefits, a mechanism understood as public money flowing into private hands because of the chronic shortage of social housing (Cole et al., 2016). Criticism was directed at absentee landlords (those known to be living out of the area) who exploited low property prices and neglected maintenance, putting tenants’ health and safety at risk, thus linking wealth extraction to ill-health and inequalities driven by government policy.

“Private landlords are just buying them up though. You will get some good, alright private landlords, I’m not knocking that, but generally, it’s just a profit-making industry, isn’t it? They don’t do any repairs either, of the ones that I know. They’re not nice places to live” (Hartlepool 2).

Despite mostly negative views, a few participants shared positive experiences. One felt fortunate to have a long-term, affordable apartment. However, this sense of fortune may reflect a relative perspective, as £480 for a two-bedroom apartment could be viewed as high in comparison to social housing options. While social housing was described as nearly inaccessible due to long waiting lists, the average rent for a three-bedroom semi-detached house in Blackpool was recently reported at £400 per month (Parkinson, 2024).

“I’m in a two-bedroom apartment. I’m very lucky. I only pay something like £480 a month, which is good. I’ve been there 10, 12 years, she only charged me for a one-bedroom at the time I wasn’t working and I was on benefits and stuff. For me to move, it would cost me, probably, an extra £150 a month, really. I’m lucky. I’ve got a good landlady. She even wrote to me last month saying, “I’ve got to put it up a tenner a week,” and I’m like, “Well, you’ve never put it up since I moved in 12 years ago” (Blackpool 2).

Overall, participants described private landlords as prioritising profit over property maintenance, with many stories of damp, mouldy homes being common and some extreme cases of no hot water or heating. This was seen as exploitation which physically harmed tenants in the pursuit of profit, as many of these properties were considered uninhabitable and detrimental to health.

“What tends to happen is that we have, not all, but quite a few private landlords who don’t provide the quality of housing that any decent human being deserves … why don’t they invest in their properties? Because they don’t have to … there is no requirement to invest … there is no housing standard for the private rented sector … there are a set of safety standards, but I summarise it as ‘try not to kill your tenants’ that’s about it. There’s no space standard, there’s no hygiene standard, provided the gas fire isn’t emitting carbon monoxide and you can get out if there’s a fire, that’s it” (Blackpool 3).

“There are thousands of people that I think are being overcharged and living in squalor. The amount of people that we work with that are getting poorly because of mould, and damp, and this, that, and the other, and nothing ever happens, and it’s, like, surely that shouldn’t be allowed. And we have an extreme lack of social housing, which is why there’s so much private rented” (Torbay 2).

Participants working in support services, conducting home visits, were shocked by the poor living conditions tenants endured. They encountered homes where children lived in rooms with leaks, water running down walls, and widespread black mould, all contributing to residents’ ill-health. Despite recognising the need for intervention, they felt powerless, as these homes were privately rented and depended on landlords taking action. One participant described tenants living without water or electricity, while another reported raw sewage leaking down walls daily due to a broken toilet that the landlord refused to repair.

“We went into some properties as well because people would invite us in to say, “I’ll show you this black mould. I’ll show you this damp. I’ll show this massive hole,” like one woman, I’ll never forget, showed me a hole in the ceiling that went up to the bathroom that the landlord wouldn’t fix. The toilet, every day, basically, poo came down the wall and she’d have to plunge the toilet every day because it was broken. It was just horrendous” (Blackpool 4).

Some participants shared personal experiences with negligent landlords. One had lived for years with a collapsed ceiling, a damaged kitchen wall, and no central heating, yet the landlord failed to make repairs. Another was told to *“boil a kettle”* after reporting a lack of hot water. These participants, and many others, described landlords as primarily focused on profit often opting for cheap, temporary fixes like painting over damp patches rather than addressing the underlying issues.

“Part of the ceiling, been down three years, the kitchen wall fell down, that’s been like that two years. He’s never dealt with it. I said to him, "Hey, come on, we’ve got no central heating in our flat." (Torbay 3).

“I had an awful landlord in the other place, I had no heating, no hot water, and he said, “Boil a kettle.” I ended up putting heating, a new thing in, myself and then he decided to sell so … I mean I got it all lovely and then … So the landlords here aren’t very nice … they just want the money … If you’ve got damp they’ll say, “Oh, we’ll just paint over that.” Anything to save money.” (Hastings 2).

Current legislation offers tenants little power to challenge negligent landlords. Participants described how tenants feared complaining to local authorities about their landlords’ inaction, worried about the risk of receiving a no-fault eviction notice. A threat some participants had personally faced after council enforcement officers intervened on their behalf. Although the new Labour government has introduced legislation to ban no-fault evictions it is not expected to take effect until at least summer 2025 (Cromarty and Barton, 2024). Commonly known as a Section 21 notice in England, a no-fault eviction notice allows landlords to evict tenants without providing a reason, exacerbating feelings of insecurity and reinforcing the power imbalance between landlords and tenants.

“If they want you out, it’s easy because of section 21. They just say “oh your rent has gone up 500 quid now” or “I’m kicking you out because I want to do something different” (Hartlepool 3).

“So they go into houses that are privately-owned and are not maintained to the standard they should be. We were so glad – “Oh, this is great, this is …” So if you’ve been complaining about damp in your house or your windows don’t fit properly, now you’ve got two guys from the council saying, “Let me look at that, because I can help with that, I can get in to do it.” So they’re going in, they’re saying to the landlord, “I want this, this, this, this, this done, you’ve 28 days to do it. This is not fit for this family.” So the guy thinks [landlord], “Stuff that,” and gives them notice to quit in 28 days. Now we’ve got families, people, all over the place with no-where to go … section 21 because they complained … on my street 11 people got section 21 … so what people are saying is when the teams [council] come round to your door and knock on your door and say “we’ve come to inspect” don’t let them in cos you’ll get section 21” (Blackpool 5).

Participants reported incidents where families were being served with eviction notices so landlords could rent their properties to the local authority. Landlords would then receive a higher, guaranteed rental income, regardless of whether the council placed a tenant in the property. Under this arrangement, the council leases the property from the private landlord for a fixed term, often up to five years, paying a guaranteed monthly income and covering any tenant-related damages (City Borough Housing, 2023).

“One of things we’ve noticed with some of our families is the private landlords are … The tenants, they’ve been great. The families, they’ve been great, they’ve been good tenants. All of a sudden the private landlords are terminating their leases. What they’re doing is they get more from the council. If they’re housing people for the council they get paid more. They’re making people homeless, people are then having to go to the council. They’re not coming back to them [houses] necessarily, but they’re taking somebody off the council list and the council pay more than they’re getting. I didn’t realise that. I’ve had two families that have told me that” (Hastings 3).

When tenants receive eviction notices, finding alternative accommodation is especially challenging due to the lack of social housing in these towns. Participants described how they, or people they knew, had presented as homeless to their local council only to be offered tents or accommodation in other towns, far from their social networks.

“We see people coming out of prison getting given a tent and a sleeping bag because the accommodation is non-existent” (Torbay 4).

“My friend [redacted], we lived in the same road, when he split up with his wife he had to leave because the rent was too high on his own. The council offered him a tent.. he’s in his 60s, he’s had 2 heart attacks, and they offered him a tent … my previous landlord did the same, he was selling up so I went to the council and they said all they could offer was a women’s refuge in London, they had no council houses. Luckily my daughter had space for me until I found this place” (Hastings 4).

Housing insecurity, whether the threat of eviction or being evicted, is linked to poor physical and mental health (Smith et al., 2024). One participant shared the intense stress of receiving an eviction notice, expressing anger and visible distress at the council’s response. Seeking housing assistance for themselves and their young son, they were told that their debts and lack of a reference from their previous landlord likely rendered them ineligible for the council house waiting list. Instead, they were advised to explore alternative private rental options.

“They told me, “you’re going to have to go into private” and I said “I won’t go into private, if that can happen to me after 12 years of paying my fucking rent month in, month out, leaving houses in a better standard than I fucking found them in, if that can happen to me, why would I ever go back into private rented?” And I said, “I’ll go sleep on my mates couches before I do that. Me and my son will sleep on couches before I do that” you know what they said to that? “oh right, so you’re saying actually you can find yourself suitable accommodation?” I said “fucking suitable? How is couch surfing suitable?” they said it’s suitable “you’re telling me you can go and stay on a friends’ couch” (Blackpool 6).

Sadly, a friends’ couch, a tent, and a domestic abuse shelter 100 miles away from your social networks, were considered suitable options for those evicted from their homes so landlords could generate more wealth. As one participant put it, private landlords were seen as profiteering from the property-less, with the sentiment summed up as, *“they’re benefitting from our misery”* (Hartlepool 4).

### 3.3 Houses in Multiple Occupation (HMOs)

Profit maximisation by private landlords was most evident in participant narratives about Houses in Multiple Occupation (HMOs). HMOs, defined in the Housing Act 2004, are legal but regulated rental properties occupied by three or more tenants forming two or more separate households, who share facilities such as kitchens or bathrooms (Housing Act, 2004). Mandatory licensing applies to HMOs with five or more occupants forming multiple households. These licensing rules are enforced by local authorities, but enforcement is uneven regionally due to limited resources. Many non-compliant HMOs are neither inspected or fined, meaning landlords often avoid penalties even when required to be registered and manged according to statutory standards (HM Government, 2021). Discussions about HMOs were more common in Blackpool and Torbay, reflecting their higher concentrations of former hotels, guesthouses and bed & breakfasts (B&Bs) that are readily available as multiple units.

“I think it became popular in Victorian times, that’s when all the big houses were built, that are now HMOs, run down and filled with mould. Some of the beautiful buildings that we’ve got, that are just so rundown … One of the sad things in these is that the agencies buy these places, carve them up into four or five bedsits, and then put people in there that have got real vulnerability … We just heard that some of the HMOs, that can’t get people in because they’re so dire, are advertising in prisons” (Torbay 5).

“I’m sure you’ve been told, but it’s about the fact that one of the symptoms of being a declining coastal town was the availability of hotels and bed and breakfasts, which were then turned into houses of multiple occupancy” (Blackpool 7).

HMOs were easy to spot around the towns, during observations wandering around these coastal streets the telltale signs became apparent; multiple doorbells, refuse bins, and satellite dishes were a common sight. Initial speculation about these properties being HMOs was confirmed by participants and informal conversations with residents.

“I noticed a house, what looked like a house not an old B&B or guesthouse, it looked like a standard terraced property but it had three satellite dishes on the outside, three wheelie bins, and three doorbells. A neighbour was sat on a doorstep smoking a cigarette, she looked at me suspiciously so I said hello hoping it would lead to a brief conversation, but also to genuinely ease her apprehensive gaze. She asked me if I was lost but I nervously laughed – my standard approach for calming a tense situation – told her I didn’t quite know where I was as I lived in the North East but explained what I was doing wandering the streets of Blackpool. I asked about the multiple satellite dishes and she confirmed that the house had been split into three flats, she said houses were really cheap here and you could make even more money by “chopping them up” and “stuffing more people in them” (Fieldnotes: Blackpool 31.10.23).

Participants largely viewed HMOs negatively, describing landlords converting cheap properties into multiple units for even greater profits than renting as single home. The introduction of Local Housing Allowance rates in 2008 is believed to have provided financial incentives for landlords to convert properties into HMOs (Smith, 2012). Participants also noted that individuals or investment organisations, often from London or beyond, were responsible for buying up properties and converting them into HMOs in their towns.

“These landlords from London, investors, bought them up, carved them up into five or six HMOs, had to fill them. They advertised in prisons, and people came out of institutions, or their marriages broke up, they drifted into Torquay, and it’s really cheap here.” (Torbay 6).

Smith (2012) argues that HMOs contribute to the clustering of single young adults on housing benefit, which partly explains persistent socioeconomic deprivation in coastal towns. Participants echoed this view, describing HMOs as primarily housing single adults, people with mental health issues, or those struggling with drug and alcohol problems, which they believed attracts criminal activity. They also noted that this influx of vulnerable individuals placed greater pressure on local services, making it harder for them to access doctors or dentists, and caused financial strain on local authorities.

“They started buying up houses for people with mental health issues, and drug and alcohol, and learning disabilities. You’re cramming vulnerable people in together. More recently you get the county lines, and then you’re getting the street drug dealers … there’s just lots more demand on the local authority for stuff, doctors and dentists, don’t even get me started on dentists I haven’t even got one, you can’t get one.” (Torbay 7).

This increased financial strain, combined with reductions in centrally provided government grants (funding allocated by national government to local government to support public services) (Alexiou et al., 2021), were suggested as the main reasons these towns are among the most deprived in the country. Participants argued they saw outward migration of healthy, skilled, and talented residents to more affluent areas with better employment opportunities, and in return received inward migration of vulnerable people suffering ill-health without the commensurate budgets to adequately support them.

“What we see, and the best way of describing it, I guess, is we import ill health and export good health. The reason for that is our housing, so our housing issue is because we’ve got cheap HMOs” (Blackpool 8).

Coastal towns were seen to naturally attract vulnerable individuals, one participant put it simply: *“if you’re going to be homeless where better to be homeless than the seaside, lots of passing trade for begging and it’s also a beautiful place”* (Torbay). However, they also described how other local authorities were moving people into these areas, seeking accommodation for those being displaced by rising rents and a lack of social housing in their own towns. This forced migration was discussed by participants in all four towns.

“there’s been a big thing around here, because of cheap housing, people who are homeless or in need of housing are being encouraged to move out of their areas, they’d literally just been, someone got a removal truck for them and maybe they’d been given an incentive of some white goods or whatever, and just being moved into a private rented property here. It’s a really huge problem … that’s driven by a lack of housing elsewhere in the country, cases coming up from London and just being moved into an area they’ve never heard of” (Hartlepool 5).

## 4 Discussion and conclusion

Empirical research on health inequalities has traditionally focused on horizontal analyses examining relationships between people, places, behaviours and community environments. While valuable, this approach can obscure the vertical political and economic structures that shape and perpetuate health inequalities. The relative absence of vertical analyses has been criticised as enabling the persistence of a health inequalities industrial complex, which depends on the continuation of inequalities to justify its existence (Ezell, 2024; Mrig and Spencer, 2024).

This study illustrates the importance of scaling up our analyses to expose the systemic mechanisms that perpetuate health inequalities. Housing, a key social determinant of health, has frequently been examined through a horizontal lens, focusing on internal conditions, tenure, or area characteristics (Gibson et al., 2011), this perspective risks overlooking policy decisions, economic structures, and governance practices, that underpin unequal outcomes. By highlighting these vertical processes, this study contributes to a growing literature that conceptualises housing not merely as a contextual factor, but as an active mechanism of inequality production (Hochstenbach, 2025; Rolfe et al., 2020; Smith and Stewart, 2024). These findings align with Fundamental Cause Theory (Link and Phelan, 1995; Phelan et al., 2010), which emphasises the role of flexible resources (wealth, security, power) in mitigating risk.

In the towns studied here, housing shaped access to such resources; insecure tenancies, poor conditions, and residualised social housing limited residents’ ability to control their environments and protect their health. These mechanisms reflect a broader political economy in which housing operates as a conduit for the unequal distribution of risk and resilience. Fundamental Cause Theory helps to frame these patterns as structural rather than circumstantial, and resistant to superficial or localised interventions.

Market-driven housing policies, particularly Right-to-Buy, have facilitated the transfer of social housing into the private sector, reducing the stock of affordable homes and exposing residents to precarious and often harmful rental conditions. This shift has had acute effects coastal towns, exacerbating financial strain, insecurity, and poor living environments, factors associated with adverse health outcomes (Rolfe et al., 2020). Health inequalities, therefore, persist not solely because of individual behaviours or localised social determinants, but are rooted in structural factors (Bambra et al., 2019; Marmot and Bambra, 2024; Sayer and McCartney, 2021). Participants’ perspectives align with existing literature into lay explanations of health inequalities, identifying housing as a horizontal social determinant (Davidson et al., 2006; Garthwaite and Bambra, 2017; Popay et al., 2003), and expand their narratives vertically in line with more recent evidence (Bernard et al., 2024; Mackenzie et al., 2017; Smith and Stewart, 2024; Smith and Anderson, 2018) to provide a more complex and nuanced understanding illustrating the interplay between horizontal and vertical factors which produce and maintain health inequalities. While they described poor conditions and insecurity, they also identified vertical drivers including Right-to-Buy legislation, no-fault evictions, weak regulation, and a policy environment that incentives private landlords. These findings reinforce the need to integrate horizontal analyses of community-level determinants with vertical examinations of systemic forces (Bambra et al., 2019; Bernard et al., 2024).

Furthermore, this study adds to scholarship framing housing within the political economy of health. Recent work by Harris and McKee (2021) and Hochstenbach (2025) has argued that research on housing and health is dominated by horizontal analyses, obscuring structural logics, such as marketisation, financialisation, and the retreat of the welfare state, that shape health outcomes (Harris and McKee, 2021; Hochstenbach, 2025). Viewed through this lens, housing can be seen as an extractive industry. Rising rents, evictions, and substandard conditions harm health directly. Since 2007, the number of people living in privately rented accommodation has nearly doubled to 4.4 million by 2021 (HM Government, 2022). Yet, many of these properties fail to meet basic standards due to weak regulation and fears of eviction (Rugg and Rhodes, 2018). These poor conditions are linked to respiratory diseases, mental health issues and premature mortality (Bambra et al., 2010; Gibson et al., 2011; Shaw, 2004) costing the NHS an estimated £1.4 billion annually (Garrett et al., 2021).

Neoliberal housing policies have privatised social housing, reduced access to quality affordable homes, (Glynn, 2009), and redirected public funds, via housing benefit, into the private sector rather than expanding social housing stock. While increasing welfare benefits for low-income groups has been shown to reduce health inequalities (Bambra, 2022; Simpson et al., 2024), this approach overlooks the economic relationships between poorer and affluent groups (Sayer and McCartney, 2021). In the context of housing, housing benefit payments illustrate how wealth is transferred from the government to private landlords through propertyless tenants. Between 2021 and 2026, it is estimated that the government will transfer over £70 billion to private landlords via housing support, more than six times the amount allocated for building affordable housing during the same period (New Economics Foundation, 2024). These dynamics exemplify how state support mechanisms, intended to aid low-income households, can entrench structural inequalities (Bambra et al., 2019; Coburn, 2004; Sayer and McCartney, 2021). Jacobs and Manzi (2017) argue that focusing on demand-side solutions like housing benefits, without addressing supply-side issues, investment in social housing, perpetuates wealth extraction and neglects attending to the vertical drivers. They emphasise a lack of political will to challenge market logics or pursue direct public provision (Jacobs and Manzi, 2017). This reflects broader commitment to neoliberal governance, where the state’s role is limited to subsidising access to housing through demand-side mechanisms, rather than intervening directly in the housing market through redistributive investment or expanded public provision.

Addressing housing-related health inequalities therefore requires comprehensive policies that engage with both horizontal and vertical dimensions. Enhancing tenant protections, investing in social housing, and redistributive policies targeting wealth accumulation in the housing market are essential. This study underscores the need to ‘scale up’ analyses of health inequalities moving beyond mitigating local harms to confronting structural forces that produce them.

## Supplementary Material

Supplementary data to this article can be found online at https://doi.org/10.1016/j.socscimed.2025.118707.

## Figures and Tables

**Table 1:** Area profiles developed using https://www.ons.gov.uk/visualisations/customprofiles/build/and Camacho et al. (2024).

Town, English Region	Population size	Demographic	Present Economy	Health outcomes
**Hartlepool, North East**	92,300	Ageing population	Deindustrialised. Largest industry health and social care.	Health overall worse than national average. 3rd Highest in England for deaths by drugs alcohol and suicide.
**Blackpool, North West**	141,000	Ageing population	Deindustrialised. Largest industry health and social care.	Health overall worse than national average. Highest in England for deaths by drugs alcohol and suicide.
**Torbay, South West**	139,300	Ageing population	Deindustrialised. Service industry now dominates.	Health overall worse than national average. 13th in England for deaths by drugs alcohol and suicide.
**Hastings, South East**	91,000	Ageing population	Deindustrialised. Largest industry health and social care.	Health overall worse than the national average. Ranks 70th for deaths by drugs alcohol and suicide.

**Table 2:** Data collection methods by field site.

Data collection method	Hartlepool	Blackpool	Torbay	Hastings	Total
**1-2-1 interview**	24	18	23	21	86
**Dyad interview**	0	1	2	2	5
**Walking interview**	0	1	2	0	3
**Focus group**	2	2	4	2	10
**Total**	26	22	31	25	104

**Table 3:** Participant characteristics by field site.

Field site	Professional	Professional resident	Voluntary sector	Voluntary sector resident	Resident	Total
**Hartlepool**	3	6	2	4	19	**34**
**Blackpool**	3	3	2	10	12	**30**
**Torbay**	0	6	0	11	25	**42**
**Hastings**	4	4	0	9	17	**34**
**Total**	**10**	**19**	** 4**	**34**	**73**	**140**

## Data Availability

The authors do not have permission to share data.
